# Linking the Microstructure of Ball-Milled Mg–Ni Hydrogen Storage Materials to Reactive Properties and Techno-Economic Feasibility

**DOI:** 10.1021/acs.energyfuels.5c01986

**Published:** 2025-07-07

**Authors:** Haoliang Hong, Alexander R. P. Harrison, Binjian Nie

**Affiliations:** Department of Engineering Science, 6396 University of Oxford, Oxford OX1 3PJ, United Kingdom

## Abstract

Solid-state metal hydride hydrogen storage exhibits advantages compared to gaseous or liquid storage, including high volumetric hydrogen storage density and improved safety. However, challenges related to technological and economical scalability, including kinetic and thermodynamic limitations, cyclability, and cost concerns, remain unresolved. In this work, Mg–Ni composites were synthesized by ball milling to identify the effects of milling parameters on performance. The macro- and microstructures of the materials and hydrogen absorption properties were investigated to assess performance for hydrogen storage. Additionally, techno-economic analysis was conducted to evaluate feasibility for practical applications and the relative effects of synthesis conditions on overall cost-effectiveness. The results indicated that variations in milling time and rotational speed modified lattice parameters and particle sizes, which in turn influenced hydrogen absorption behavior. From the techno-economic analysis, a ball milling time of 2 h at 300 rpm speed produced the most cost-effective material in terms of balancing total capacity and electricity costs (0.77 $ per kg H_2_ stored).

## Graphical abstract

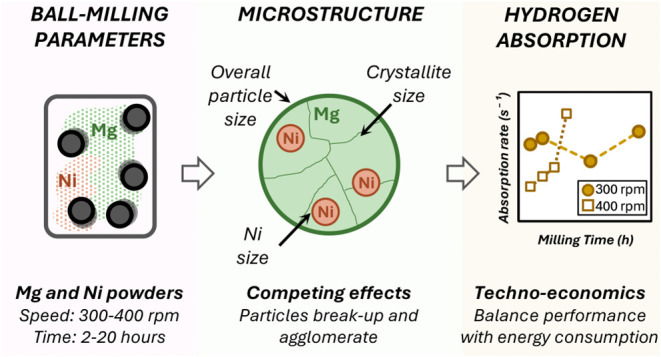

## 1 Introduction

Amid the growing demand for energy and worsening environmental conditions, countries and international organizations are increasingly recognizing the importance of transitioning to green energy. Among the 17 sustainable development goals (SDGs) proposed by the United Nations (UN) for 2030, seven concern the practical use of clean energy technologies, while the European Union (EU) has set a target of achieving carbon neutrality by 2050.^1,2^ Hydrogen, as a clean energy carrier with high energy density relative to battery systems,^3,4^ is a pivotal resource for addressing global warming, and plays a crucial role in advancing the transition away from fossil fuel dependence.^5,6^ Despite showing some promise, safety concerns and cost issues, especially regarding hydrogen storage and transportation, severely impede the development of the green hydrogen industry.^7–9^ Consequently, it is crucial to move beyond existing technologies such as liquid and gaseous H_2_ storage and toward novel technologies and materials with readily scalable production methods. Solid hydrogen storage, in the form of hydrogen absorption to form metal hydrides (shown in eq 1, for magnesium-based hydrogen storage allows), or hydrogen adsorption onto functionalized nanoporous carbon,^10–12^ helps address these problems by offering intrinsic safety, as the H_2_ is stored as a relatively inert solid, which significantly reduces the risks of leakage, high-pressure hazards and uncontrolled combustion, while achieving high volumetric H_2_ density.

1 $$\mathrm{Mg}+H_{2}↔\mathrm{Mg}H_{2}$$

Magnesium-based hydrides exhibit robust stability over repeated cycles of hydrogen absorption and desorption without losing capacity,^13^ and high gravimetric hydrogen capacity (up to a theoretical maximum of 7.6 wt %). By the addition of transition metal catalysts (e.g., Ti, Fe, Ni) to aid hydrogen dissociation,^14–16^ and incorporation of graphitic carbon to aid heat transfer during hydrogen absorption and desorption,^17^ Mg-based alloys have been applied successfully in pilot scale hydrogen storage systems (10–1000 kg stored H_2_).^13,18^ However, two major challenges remain: high reaction temperatures and slow hydrogen sorption kinetics. Forming alloys or composites of Mg with catalytically active elements decreases the reaction temperature and pressure, but can result in the formation of metastable hydrides with lower enthalpy (Δ*H*).^19,20^ To mitigate this problem, catalyst addition, using transition or rare-earth elements (e.g., Ni), lowers activation energy and facilitates hydrogen dissociation.^21–24^ Nanosizing the material particles also reduces diffusion distances and increases surface area, accelerating hydrogen ab/desorption.^25–27^

High-energy ball milling is a widely used technique for fabricating Mg-based alloys, as it enables particle size reduction, strain hardening, and atomic-scale mixing.^18,28^ The process also introduces lattice defects via severe plastic deformation, which serve as diffusion pathways for hydrogen, thereby improving storage kinetics,^29,30^ hence reducing the required reaction pressure and temperature.^31^ However, when scaled up for industrial applications, optimizing economic efficiency requires balancing gains in hydrogen storage against the costs of ball milling. Other studies have investigated the use of novel fabrication techniques including direct current arc plasma^32^ to deposit Mg-based composite nanoparticles onto an oxide support (e.g., Al_2_O_3_, TiO_2_, Fe_2_O_3_), or molten metal atomization.^33^ However, despite initial promising results in terms of hydrogen capacity and reaction rates, both processes are relatively novel compared to ball milling, with further development required in order to be applied at a practical scale for the industrial production of Mg-based materials for hydrogen storage. Furthermore, Mg-based materials prepared via atomization have also shown suboptimal stability, as a result of Mg oxidation to MgO during alloy production.^34^ For production of Mg-based materials at very large scales (>100 kg), where the energy costs associated with high-energy ball milling become prohibitive, and to avoid agglomeration and oxidation during milling,^35^ alternative mechanochemical preparation methods have been investigated, including fast-forging and high-pressure torsion of Mg-based materials,^36,37^ albeit with a multistep process of forging and annealing required to achieve a product microstructure and phase composition capable of fast absorption and desorption of hydrogen.^37^

The effects of ball milling parameters on the structure and hydrogen storage properties of Mg-based materials remain unclear. Some studies indicate that prolonged milling time is beneficial for improving hydrogen storage, as seen in Mg_1.95_Y_0.05_Ni_0.92_Al_0.08_ (240 h milling exhibited twice the cycling performance than 12 h^38^) and LaMg_11_Ni + 100 wt % Ni (60 h milling was 1.5 times the capacity compared to 20 h^39^). Others report optimal hydrogen capacity at shorter durations, such as La_7_Ce_3_Mg_80_Ni_10_ (optimal hydrogen storage capacity at 10 h milling in 0–30 h range) and Ce–Mg–Ni (peak at 5 h between 5 and 20 h milling), due to reduced particle agglomeration.^40,41^

The objective of this study is to investigate the effect of ball milling parameters (rotational speed and milling duration) on the hydrogen storage properties of Mg–Ni-based materials. The effects of milling conditions are correlated to the microstructural properties of the materials and then linked to the rates of hydrogen storage and discharging and total hydrogen capacity. Therefore, our research can help optimize the production process for solid-state hydrogen sorbents, reducing the amount of wasted materials and energy, and improving the net efficiency of future hydrogen storage systems.

## 2 Experimental Section

A series of Mg–Ni binary metal composites were prepared by high-energy ball milling using a planetary mill (Pulverisette 6 Classic Line Planetary Mono Mill, Fritsch), composed of a 250 mL zirconia milling jar and 50 zirconia milling balls with a total weight of 200 g. Pure Mg powder (9 g per batch; 99.8% purity and 325 mesh particle size, Fisher Scientific) and Ni powder (1 g per batch; 99.8% purity and 300 mesh particle size, Fisher Scientific) were used as the starting materials, with a constant 20:1 ball-material mass ratio and a 9:1 Mg–Ni mass ratio.^42^ The milling speed (300 and 400 rpm) and the total milling time (2, 4, 6, 8, 12, and 20 h) were varied for each sample, described in the Supporting Information (SI),Table S1, with samples designated as Mg_90_-[Speed]-[Time] (e.g., Mg_90_-300–2h for the sample milled at 300 rpm for 2 h).

To prevent excessive heating, resulting in welding and agglomeration of metal powders, the ball milling was stopped every 30 min, allowed to cool for 10 min, and repeated until the material had been milled for the overall target time. Metal precursors and milled samples were stored in an Ar-filled glovebox to prevent surface oxidation in air. For material characterization and hydrogen absorption and desorption experiments, the entire distribution of particle shapes and sizes was used, obtained directly from the milling jar. Although size screening has been commonly employed in previous studies,^43^ the present work placed particular emphasis on economic considerations, and consequently, bulk samples that represented the ball milling process only, without further processing, were favored. For experiments determining the intrinsic thermodynamic and kinetic properties of Mg_90_-300–2h, the sample was sieved to <150 μm, to remove any uncertainty induced by a broad distribution of particle sizes.

A series of characterization and measurement techniques were applied for each sample. Particle size distributions for each sample were estimated from images (shown in the SI, Section B) collected using a bright-field optical microscope (MT5000 Series, Meiji Techno). Surface elemental distributions for each sample were estimated from scanning electron microscopy–energy-dispersive X-ray spectroscopy (SEM-EDS) images, collected using a JEOL IT800 microscope equipped with a JEOL energy-dispersive spectroscopy (EDS) detector. All image analysis was performed using ImageJ software.^44^ X-ray diffraction (XRD) patterns were collected using a Bruker D8 diffractometer using Cu Kα radiation at 40 kV and 40 mA, with a measurement range of 2ϑ = 10–80° and a step size of 0.02°. Instrumental peak-broadening parameters were determined by measurement of a corundum standard (NIST, Standard Reference Material 1975b)^45^ with subsequent Rietveld refinement. Refinement was performed using GSAS-II software,^46^ using reference phases from the ICSD database^47^ (entries with collection codes Ni: ICSD8688, Mg: ICSD29734, Mg_2_Ni: ICSD162120). Given suspected preferential crystallite orientation within metal samples as a result of processing,^48,49^ Le Bail refinement^50^ was performed to estimate lattice parameters and crystallite size, without refining phase compositions or crystallographic texture.

The Brunauer–Emmett–Teller (BET) surface area^51,52^ of the materials was estimated using a Micromeritics Gemini VII instrument, following ASTM D6556 using the 5-point BET method of N_2_ adsorption at liquid nitrogen temperature (−196 °C). Prior to experiments, the samples were degassed overnight at 160 °C until the sample mass stabilized. Material properties were calculated using MicroActive software (Micromeritics), with a further description of the data analysis given in the SI, Section C.

A Sievert volumetric apparatus,^53^ built in-house, was used to determine the gas absorption and release properties of a material by monitoring relative pressure changes within a reactor system of known volume. Hydrogen was supplied to the system using a Thales H-Genie hydrogen generator filled with deionized water (Tesco Auto, conductivity *c.* 500 nS cm^–1^), able to produce hydrogen at up to 4 MPa with nominal purity 99.99 mol %. Helium was supplied from a gas cylinder (BOC, 99.999 mol %). Since the acquired data define the pressure–composition–temperature (PCT) characteristics of the material, the apparatus is also referred to as a PCT instrument. A further description of the design and operation of the PCT instrument, and the analysis of the resulting data, is provided in the SI, Section A, with a schematic of the instrument given in Figure S1.

## 3 Results and Discussion

### 3.1 Material Characterization

Figure S2 illustrates the morphology of the samples after ball milling, and Figure 1 shows the average overall particle size, Ni particle size, and crystallite size for each sample. All samples exhibited metallic luster without any visible surface oxidation. The Sauter mean diameter ( $\overset{®}{\mathrm{SD}}$ *or d*_32_) represented the average diameter of multidispersed particle material and was computed by considering the proportion of volume-to-surface area,^54^ calculated using eq 2

2 $${\overset{®}{\mathrm{SD}}=d}_{32}=\frac{\sum_{i=1}^{n}\;d_{i}^{3}}{\sum_{i=1}^{n}\;d_{i}^{2}}$$

where *n* and *d*_*i*_ are the total number of observed particles and the diameter of each particle, respectively. All measurements for the average overall and Ni particle size, as well as their Sauter mean diameter, are reported in the SI, Tables S2 and S3.

**Figure 1:**
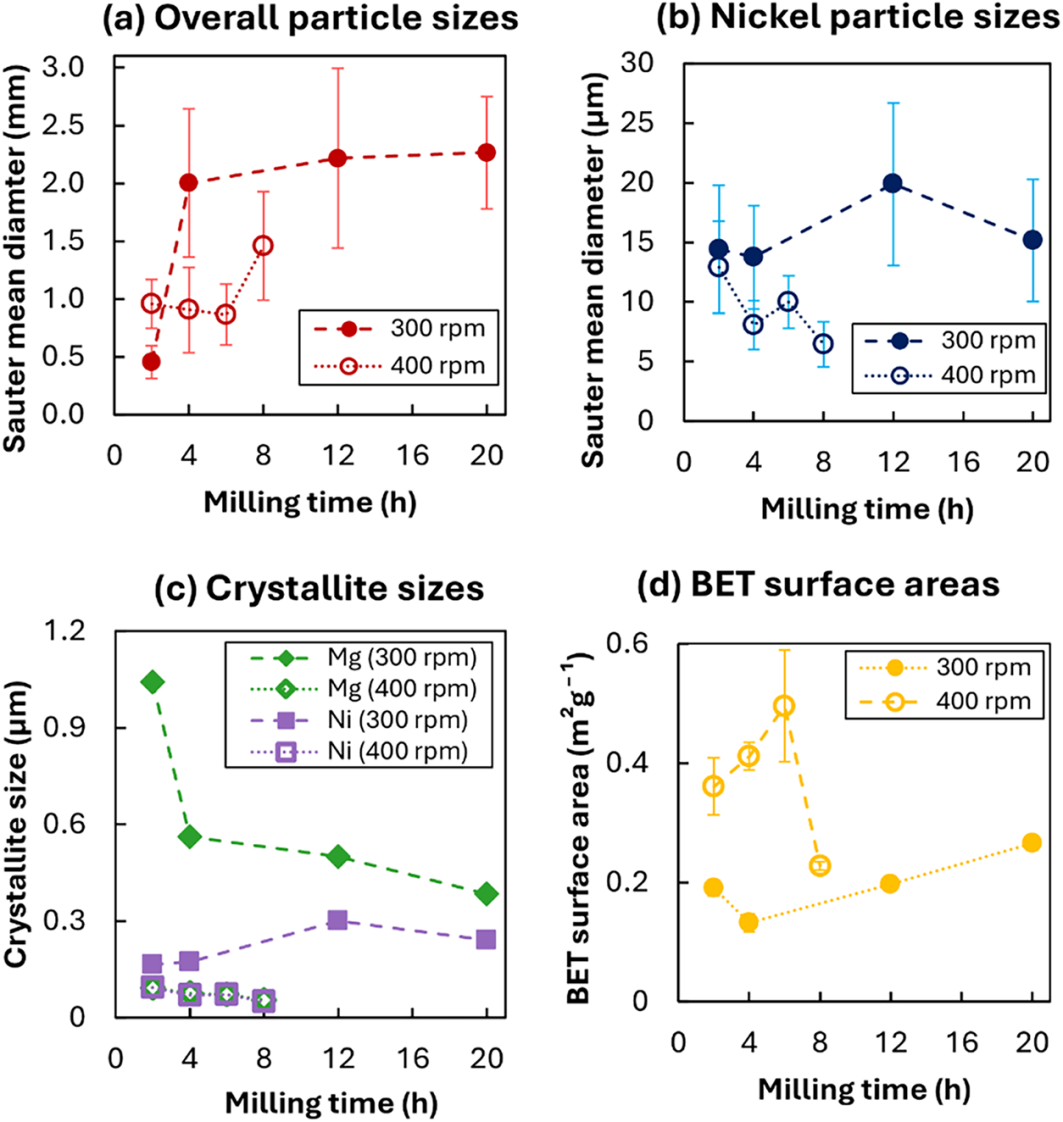
Plots for the Sauter mean diameter of (a) overall particle sizes measured from microscope images in the SI, Figure S2, and (b) nickel particle sizes from SEM images, (c) crystallite sizes of Mg and Ni calculated from XRD refinement, and (d) BET surface area estimated from N_2_ adsorption. Error bars in (a, b) indicate standard deviation in measured particle diameter; error bars in (d) indicate estimated uncertainty in fitted BET surface area.

The overall particle size of Mg–Ni ball milling products under different durations from 2 to 20 h and rotational speeds of 300–400 rpm (shown in Figure 1a) increased by 3 orders of magnitude compared to the initial powder (Mg 44 μm, Ni 48 μm). At a low rotational speed (300 rpm), the particle size increased with extended milling time; however, this phenomenon showed a marginal effect after 4 h, with average particle size >2000 μm and growing slowly. Particles milled for 2 h were smallest in size, whereas those 4 h-milled exhibited a more irregular shape and sharper edges compared to those 12 and 20 h-milled, as shown in the SI, Figure S2. At a higher rotational speed (400 rpm), the average particle sizes were smaller, with a more uniform distribution (i.e., less variation in size for each sample of particles). Compared to the overall particle sizes for samples milled at 300 rpm, the high-speed rotation caused a size reduction up to 6 h of milling. A sharp increase in average size was observed after 8 h of milling, albeit with an average size for the Mg_90_-400–8 h sample lower than all samples milled at 300 rpm for ≥4 h.

For most samples, Mg and Ni showed poor fusion, with distinct Mg and Ni particle boundaries observed. The size of Ni particles was refined from the initial value of 48 μm for the pure powder to an average below 20 μm. However, no specific trend with milling time was evident, as shown in Figure 1b. For the samples milled at 400 rpm, a longer milling time seemed to result in smaller Ni particles, but this trend was not pronounced. Nonetheless, the increase in surface roughness, composite material uniformity, as well as the fragmented and irregular features, correlated positively with milling time, visible in the Mg_90_-400–8h sample. The EDS analysis of this sample revealed a uniform and finely distributed Ni phase along with a textured composite surface that was absent for other samples. These factors may contribute to the formation of more diffusion pathways and active sites. Additional active sites could be formed by the increase in surface roughness, facilitating the dissociation of H_2_ molecules into H atoms.^55^ Grain boundaries, defects, and irregular surface structures induced by milling promoted the diffusion of H atoms, enhancing the reaction rate.^56^

The SEM images and EDS maps for all samples are displayed in the SI, Figure S3. At low rotational speed (300 rpm), the distribution of Ni was in the form of large, isolated spots for a short milling time (2–4 h), showing that the Ni had not yet been completely dispersed in the Mg. After 20 h of milling, the features of cold welding, such as blurred boundaries and embedded distribution, became prominent.^57^ However, a small number of Ni-rich regions was still observed. At a high rotational speed (400 rpm), the distribution of Ni became relatively uniform within just 2 h, indicating that high rotational speed facilitated the rapid embedding of Ni particles into the Mg. With extended milling durations (4, 6, and 8 h), the Ni distribution became further homogenized, with almost no isolated Ni-rich spots observed. Therefore, a high rotational speed (400 rpm) combined with a medium duration (e.g., 6 h) can dramatically refine and fuse particles while avoiding cold welding and agglomeration.

During ball milling, severe plastic deformation, high contact pressure, and fresh metal surface exposure can lead to cold welding of metallic powders. At low milling speeds, cold welding and fusion effects are expected to dominate over particle refinement caused by impact and shear.^56,58^ This indicated that the equalizing circularization effect cannot be achieved in a short-period of milling. The high energy generated by high-speed rotation could cause more material fusion within 2 h, however, as milling progresses, starting from 4 h, the occurrence of cold welding probably reduced. Interestingly, for samples milled at 400 rpm, short-duration milling <6 h resulted in smaller particles, with agglomeration increasing considerably after 8 h. Therefore, to achieve mechanically processed materials with the smallest and most uniform particle size, which is theoretically more beneficial for reaction with hydrogen, future process refinement could prioritize a combination of high-speed and short-duration ball milling as the optimal approach.

The XRD patterns of the ball-milled Mg90 wt %-Ni10 wt % composite materials are shown in the SI, Figure S5. All samples showed two distinct Mg and Ni phases with no evidence of an alloyed Mg_2_Ni phase.

From Le Bail refinement, the lattice parameters and mean crystallite sizes for each phase were determined. Lattice parameter, and hence unit cell volume, did not vary significantly between samples with an average unit cell volume of 43.77 ± 0.06 Å^3^ for Ni and 46.5 ± 0.08 Å^3^ for Mg, in reasonably good agreement with the reference value from the ICSD database for Ni (43.76 Å^3^), but with slight lattice expansion relative to the reference value for Mg (46.10 Å^3^).

Crystallite size (shown in Figure 1c) was found to be relatively insensitive to milling time but to be influenced by overall milling speed. For samples milled at 300 rpm, Mg crystallite size sharply decreased between 2 and 4 h of milling, then declined gradually with further milling, and Ni crystallite size remained approximately constant. For samples milled at 400 rpm, the crystallite sizes of the Mg and Ni phases were approximately equal, and did not change significantly with prolonged milling. However, both crystallite sizes were markedly smaller for samples milled at 400 rpm, with an average size of 0.08 ± 0.01 μm for Ni and 0.07 ± 0.01 μm for Mg, as compared to 0.20 ± 0.05 μm for Ni and 0.36 ± 0.08 μm for Mg for samples milled at 300 rpm. Furthermore, for samples milled at 300 rpm, crystallite size was consistently larger for Mg than for Ni, whereas, after milling at 400 rpm, the sizes of each phase were approximately equal.

For samples milled at 300 rpm, BET surface area did not change substantially with prolonged milling (area *c.* 0.14–0.20 m^2^g^–1^) shown in Figure 1d, with adsorption isotherms reported in the SI, Figure S6, with a slight increase in surface area as prolonged milling promoted particle refinement after 20 h (up to 0.27 m^2^g^–1^). Contrastingly, for samples milled at higher speed (400 rpm), BET surface area increased between 2 to 6 h (from 0.36 to 0.50 m^2^ g^–1^), followed by a sharp decrease to 0.23 m^2^ g^–1^ after 8 h of milling. The measurements therefore suggest that at high speed, particle refinement leading to higher surface area dominated initially and was then surpassed by agglomeration after prolonged milling, showing the same trend as for overall particle size shown in Figure 1a.

During ball milling, the energy transferred from the balls to the solid materials *per* collision is proportional to the square of the rotation speed, and the total number of collisions (and hence, total energy transfer) between the balls and the material being milled is linearly proportional to the milling time.^59^ Therefore, increasing the rate of energy transfer to the solids by a factor of ∼1.8 decreased the average crystallite size by factors of ∼2.5 and ∼5.1 for Ni and Mg, respectively. Hence, the results suggest that a minimum threshold of energy per collision was required to break up large crystallites, whereas transferring an equivalent amount of energy at a lower rate over a longer period (as shown in the SI, Figure S7) was insufficient to produce small crystallites. Moreover, although the formation of Mg_2_Ni is thermodynamically feasible from Mg and Ni powders, the total energy applied during milling might not have been sufficient to induce reaction between Mg and Ni. Previous studies producing Mg_2_Ni via ball milling applied milling speeds of 1100–1200 rpm,^60^ or milling times of up to 60 h,^61^ corresponding to around 10-fold greater energy transfer in each case as compared to the milling conditions applied here.

### 3.2 Hydrogen Absorption/Release and Cycling Properties

The SEM images of samples after 5 hydrogenation cycles at 2 MPa and 573 K are shown in Figure S4. Hydrogen cycling led to significant changes in the morphology of samples relative to the fresh materials (shown in Figure S2), including particle fragmentation, surface crack formation, and surface roughening. The extent of these surface modifications depended on the milling duration, with shorter milling samples resulting in rougher and more fragmented products after the hydrogen reaction compared with those milled for longer durations.

Figure 2b illustrates hydrogen uptake and release during each hydrogenation and dehydrogenation cycle for samples milled at 300 rpm, with hydrogenation at 2 MPa and 573 K, and dehydrogenation at 0.01 MPa and 623 K (temperatures selected arbitrarily, in order to compare samples with one another). Each cycle consisted of 120 min of H_2_ absorption and 10 min of desorption. For all samples apart from Mg_90_-300–12h, the first hydrogen absorption activation did not reach equilibrium within 120 min, as the amount of hydrogen absorbed did not reach a steady value, whereas for subsequent cycles, the material reached equilibrium. In the two subsequent hydrogen uptake/discharge cycles after the activation, some of the samples milled at 300 rpm showed a slight increase in the rate of H_2_ uptake.

**Figure 2:**
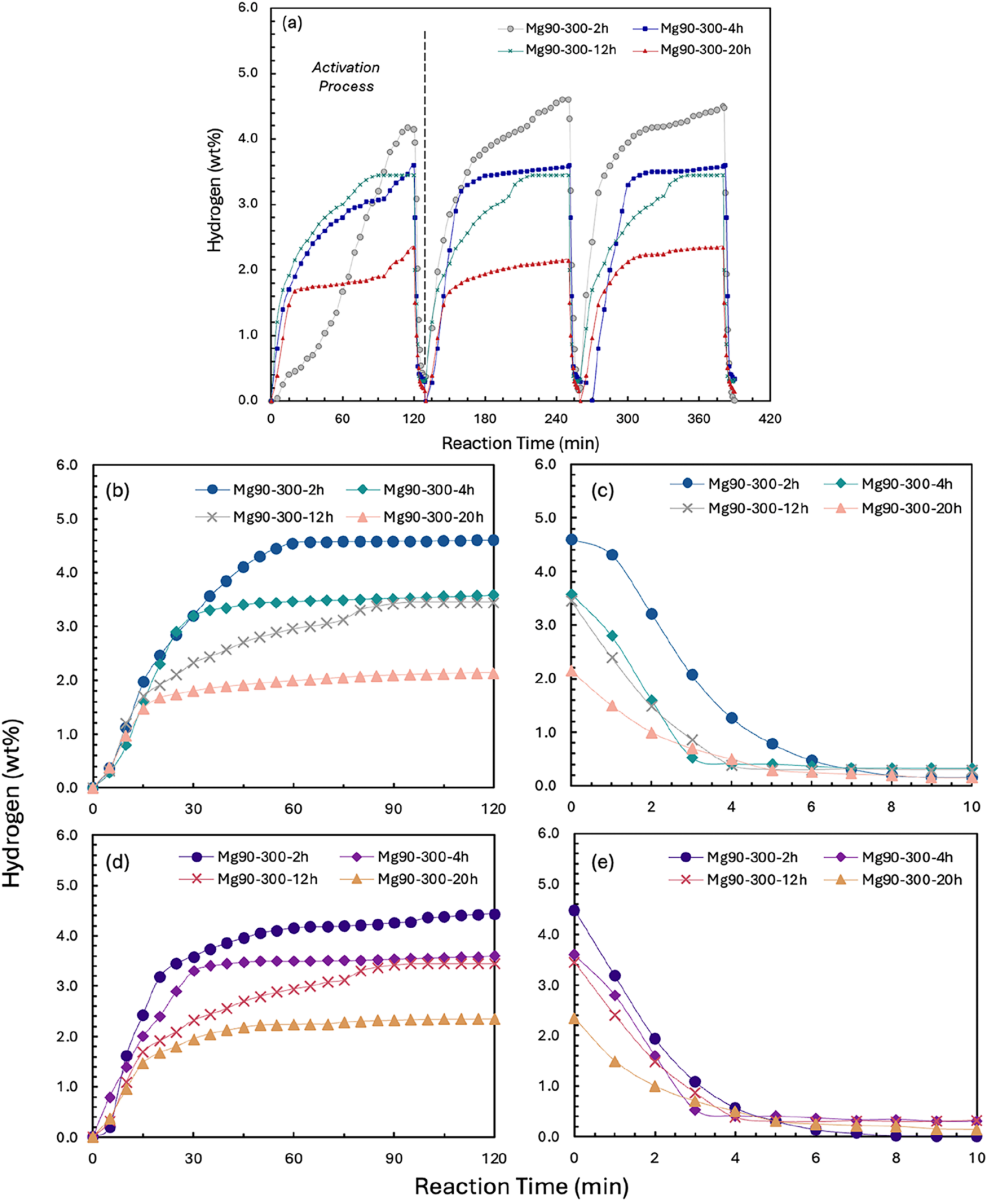
(a) First three hydrogen absorption (2 MPa and 573 K) /desorption (0.01 MPa and 623 K) loops for milled Mg90 wt %-Ni10 wt % under 300 rpm, and (b) the 5th cycle and (c) the 10th cycles of hydrogenation and dehydrogenation under the same reaction conditions.

For the second and third cycles of the Mg_90_-300–2h sample, the rate of hydrogen absorption changed during hydrogenation, with the amount of hydrogen absorbed rapidly increasing from 3.00 to over 3.40 wt % in a sharp step. The variation in absorption rate could be explained by the divergent morphologies and particle diameters (reported in the SI, Table S2), as variation in particle diameter within each could result in heterogeneous internal mass- and heat-transfer rates. In a numerical simulation model reported by Lin and Zhu et al.,^62^ the time to reach maximum hydrogen storage in metal hydrides with particle diameters of 10 or 100 μm particle diameters varied considerably (from 1500 to 3000 s). For larger particles, inward diffusion of hydrogen atoms to the core and outward dissipation of reaction heat were more challenging, resulting in slower rates of reaction. However, for the experiments reported here, these potential limitations were largely overcome through repeated cycling as no sharp increases in absorbed hydrogen were observed for the fifth and 10th cycles (shown in Figure 2b,d). Over repeated cycles, repeated stress variation as a result of volumetric expansion and contraction during the phase change Mg ↔ MgH_2_ induced particle fragmentation and crack formation (shown in the SI, Figure S4), which could facilitate heat and mass transfer.^63^ However, for large-scale applications, structural degradation might also lead to particle agglomeration over multiple cycles, reducing overall performance.

The duration of the ball milling showed an impact on the maximum hydrogen absorption capacity. The maximum hydrogen uptake of the samples milled for 2 h was the highest of all the samples, albeit with slower kinetics (i.e., a longer time required to reach equilibrium during the tested cycles), but showed a slight decrease at the 10th absorption (approximately 4.61 wt % at the second, third and fifth cycles while 4.45 wt % at the 10th cycle). These measurements of the 4, 12, and 20 h milling samples achieved 3.60, 3.45, and 2.35 wt %, respectively, and maintained recovery.

The results for samples prepared at a milling speed of 400 rpm in Figure 3 show a slightly different trend. The maximum hydrogen absorption capacity was observed after 6 h of ball milling reached 3.60 wt %, which was higher than the capacity of samples milled for 2, 4, and 8 h, at 2.61, 2.38, and 2.85 wt %, respectively. Furthermore, for samples milled at 400 rpm, a longer milling time resulted in a faster rate of initial H_2_ uptake, irrespective of the final amount of hydrogen absorbed. The increase in initial rate might be attributable to an improved catalytic effect resulting from the progressive refinement and homogenization of Ni particles, compared to samples prepared with milling at 300 rpm.

**Figure 3:**
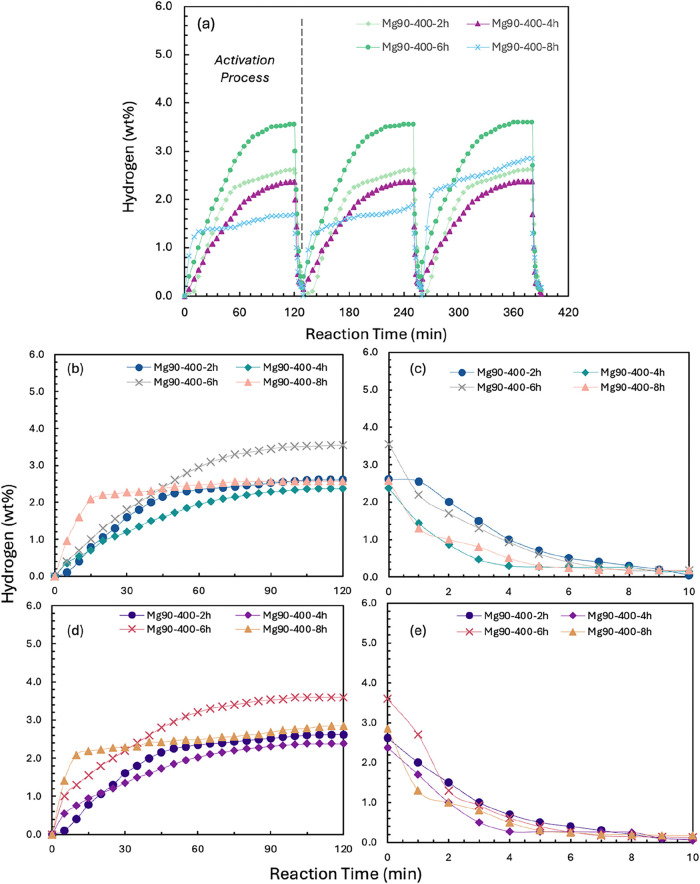
(a) First three hydrogen absorption (2 MPa and 573 K)/desorption (0.01 MPa and 623 K) loops for milled Mg90 wt %-Ni10 wt % under 400 rpm, and (b) the 5th cycle and (c) the 10th cycles of hydrogenation and dehydrogenation under the same reaction conditions.

The samples prepared at a milling speed of 400 rpm exhibited both lower maximum hydrogen absorption capacity and slower absorption rates, compared to the samples prepared at 300 rpm. This difference could be explained by the calculated crystallite sizes of Mg and Ni shown in Figure 1c, where high rotational speeds significantly reduced the crystals of both elements. A possible explanation was that excessive particle refinement may lead to agglomeration due to high surface energy,^64,65^ reducing the effective surface area, while lattice expansion and increased grain boundaries can facilitate hydrogen diffusion and trapping.^66^ Given the notably rapid uptake of the Mg_90_-400–8h sample and the similar absorption rates across samples milled at 300 rpm for 2–20 h, it is likely that 8 h of ball milling represents an optimal condition for enhancing hydrogen absorption kinetics within this parameter range.

In summary, Figure 4 illustrates the relation between two dependent variables, milling times and rotation speeds, and the hydrogen storage properties, the maximum hydrogen storage, and the absorption rate (calculated as the reciprocal of the time required to reach 50% conversion). The relationships between each individual structural parameter affected by milling times and rotation speeds (overall particle size, Ni particle sizes, and crystallite sizes) and the hydrogen storage properties are shown in the SI, Figure S8.

**Figure 4:**
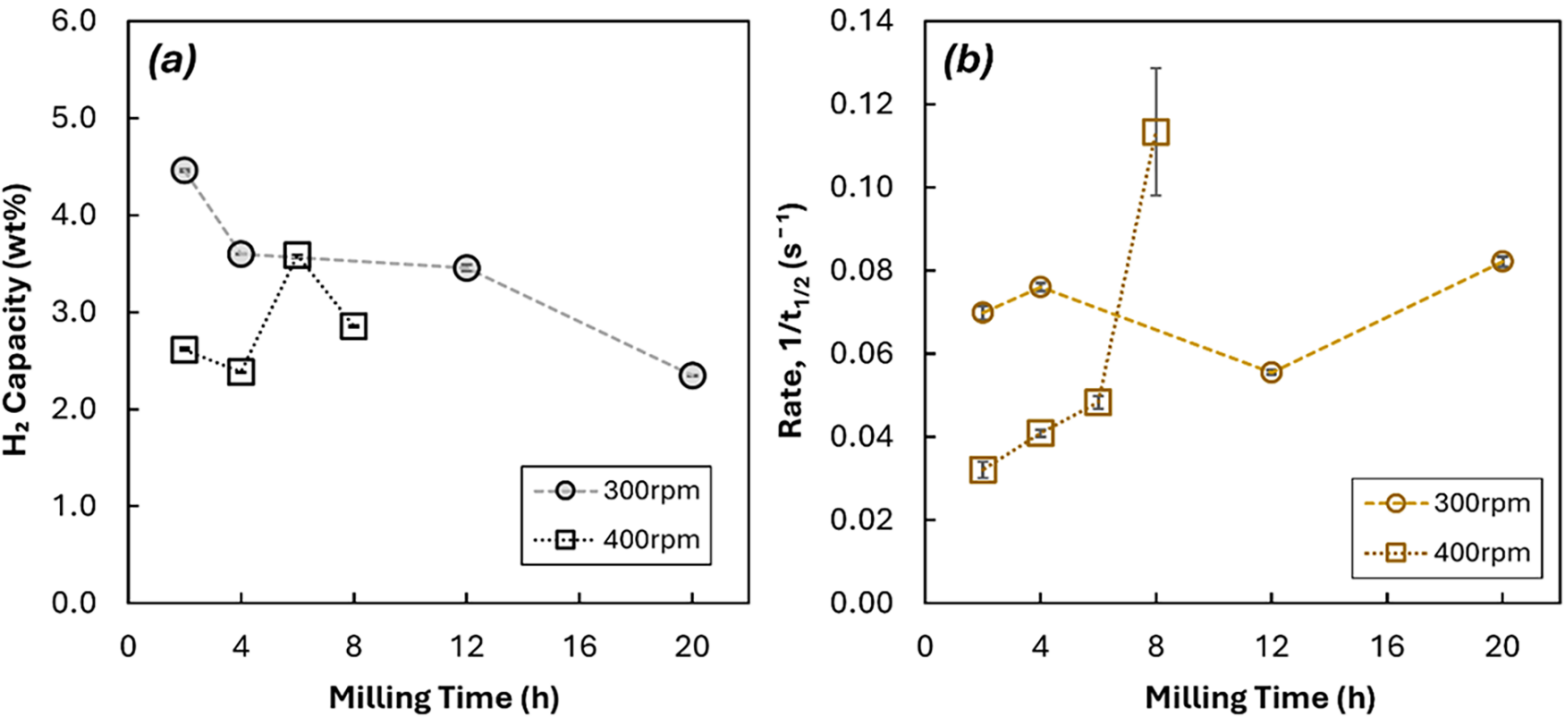
Changes in (a) maximum hydrogen storage capacity and (b) characteristic rate (defined as taken as the reciprocal of the time required to reach half of the maximum hydrogen storage capacity during absorption) for samples prepared with a variable milling speed and milling time. Error bars indicate standard deviation in measurements over three repeated hydrogenation cycles.

As observed in the SI, Figure S8a.i,a.ii, a weak negative correlation was observed between particle size and hydrogen capacity (with larger particles showing lower total capacity), and no clear correlation was observed between particle size and rate of H_2_ absorption. Similarly, little correlation was observed between Ni particle size and either hydrogen capacity or rate of absorption (shown in Figure S8b.i,b.ii). For samples milled at 300 rpm, smaller crystallites resulted in a decrease in overall capacity (shown in Figure S8c.i), and no overall change in rate of reaction (shown in Figure S8c.ii), suggesting that the increase in overall particle size counteracted any improvement to rate of reaction from crystallite refinement. Contrastingly, for samples prepared at a higher speed of 400 rpm, capacity did not show a clear trend with crystallite size, but the overall rate of reaction sharply increased for samples with smaller Mg crystallite, suggesting that increased milling time had a considerable effect on reaction kinetics through crystallite refinement, without affecting overall capacity. For samples prepared by milling at both speeds, BET surface area (shown in Figure S8d.i,d.ii) had little correlation with either capacity or rate, suggesting that any changes in surface area as a result of prolonged milling were outweighed by other factors.

Therefore, no clear trend was identified between any individual microstructural property and the overall performance. However, as shown in Figure 4, altering the preparation parameters of milling time and milling speed did influence performance, with prolonged milling at high speed (400 rpm) resulting in a considerable increase in rate of reaction. As such, overall capacity and rate of reaction are likely to be influenced by the interaction between several different microstructural properties (particle size and Ni size crystallite size). As altering milling parameters results in a change in all three properties at once (as shown in Figure 1), the results presented here allow empirical correlations to be drawn between preparation procedure and overall performance, while noting that competing effects as a result of e.g., simultaneous crystallite refinement and particle agglomeration at high milling energy might ‘cancel out’.

Future work should develop material synthesis techniques to allow each parameter to be manipulated independently, allowing stronger correlations to be identified between individual microstructural properties and overall performance, and to establish causative relationships between parameters.

Additionally, from the XRD measurements, no evidence of an amorphous phase was detected, with strong crystalline peaks and a relatively flat baseline. However, previous research has indicated that Mg-based materials with higher nickel content (up to 50 wt % Ni) can form an amorphous Mg–Ni phase,^67^ which might be able to achieve a greater effective H_2_ capacity *per* mass of Mg than the fully crystalline samples as prepared here.^68^

### 3.3 Thermodynamic Properties

Figure 5a shows the PCT thermodynamic curves for the Mg_90_-300–2h sample, with hydrogen uptake as a function of pressure and temperature. The results showed that the maximum hydrogen storage capacity increased slightly with increasing temperature, reaching 6.97 wt % at 623 K. In comparison, Hong and Lee reported that mechanically ground Mg–Ni (9 wt %) absorbed 6.33 wt % hydrogen under similar conditions.^69^ Similarly, Mg–Ni and Mg–Nd^70,71^ showed hydrogen absorption capacities of 6.79 and 6.73 wt %, respectively, as reported by Zou et al. The thermodynamic parameters for the material were calculated using the van’t Hoff equation^72^

3 $$\ln P_{H_{2}}=\frac{\Delta H}{\mathrm{RT}}-\frac{\Delta S}{R}$$

where *P*_H_2__ is the equilibrium hydrogen pressure (atm), Δ*H* is the enthalpy change (kJ mol_H_2__^–1^), Δ*S* is the entropy change (J (mol·K)^−1^), *T* is the temperature (K), and *R* is the molar gas constant 8.3145 J (mol·K)^−1^.

**Figure 5:**
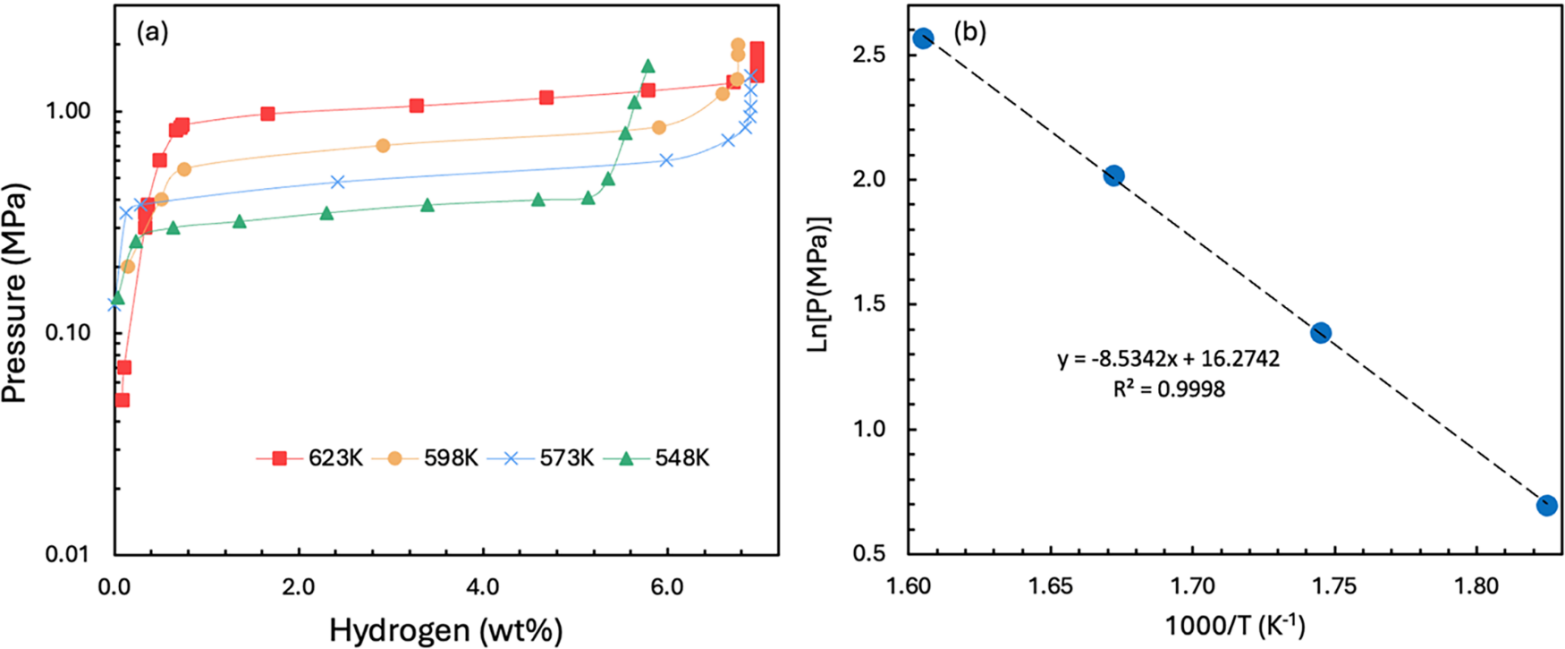
(a) PCT curves and (b) the corresponding van’t Hoff plots for Mg_90_-300–2 h.

Based on this linear relationship, the enthalpy and entropy of hydrogen absorption of the samples were obtained, giving Δ*H* = −65.94 kJ/mol H_2_ and Δ*S* = – 127.64 J/(mol·k) from the gradient and intercept of the Van’t Hoff plot, respectively (shown in Figure 5b). For comparison, the enthalpy of hydrogen absorption for pure Mg powder was −74.8 kJ/mol of H_2_. A lower absolute value of Δ*H* would reduce the dehydrogenation temperature and the amount of heat transfer required for hydrogen absorption and desorption. By comparison, pure Mg_2_Ni alloy has a Δ*H* of −64 kJ/mol H, albeit with a lower theoretical hydrogen storage capacity (3.6 wt %),^73^ indicating that the addition of Ni to form Mg–Ni composites can decrease absolute reaction enthalpy, even if the Ni remains as a separate metallic phase.

### 3.4 Hydrogenation Kinetic Properties

Figure 6a shows the rate of H_2_ uptake for the Mg_90_-300–2 h sample operating under different temperatures (518, 533, 548, and 563 K). The hydrogen absorption process of Mg-based materials generally follows a random nucleation and growth mechanism, which were modeled using the Johnson–Mehl–Avrami–Kolmogorov (JMAK) equation (eq 4) to estimate the hydrogenation activation energy.^74^

4 ln[−ln(1−α)]=ηln(k)+ηln(t)

Fitted curves of ln­[−ln­(1 – α)] vs lnt were produced as Figure 6b, where α represents the relative conversion of Mg to MgH_2_ at time *t*, *k* is the effective kinetic parameter that describes the rates of nucleation and growth (min^–η^), and η is the Avrami exponent or reaction order. From this curve, slope η and intercept η ln* k* were obtained, giving η = 0.81 ± 0.02, and hence providing corresponding values at different temperatures. Using the Arrhenius equation,^75^

5 $$k=A\;\exp\left(-\frac{E_{a}}{\mathrm{RT}}\right)$$

the hydrogenation activation energy (*E*_a_, kJ/mol) was determined, where *A* is the pre-exponential factor (min^–η^, temperature-independent). By determining the *k* values at various temperatures, a fitted curve of ln *k* vs 1000/T was plotted, allowing the estimation of the hydrogenation activation energy, *E*_a_ = 109.08 kJ/mol H_2_, and the pre-exponential factor, ln­(*A*) = 16.1. While noting that lower activation energy alone does not necessarily correspond to better performance under given reaction conditions,^76^ other Mg-based materials were reported as showing higher activation energies, such as MgH_2_ (172.61 kJ/mol H_2_), as-milled 20 h nano Mg powder (163.59 kJ/mol H_2_),^77^ as well as some Mg-rare earth systems, e.g., Mg_80_La_6.45_Ni_13.52_ (136.2 kJ/mol H_2_)^78^ and Sm_5_Mg_41_ alloy (135.3 kJ/mol H_2_),.^79^ Ruihan et al.^80^ found that short-duration ball milling had a comparable impact on the reaction kinetics and activation energy of materials. In their investigation of NdMg-Ni system, the dehydrogenation activation energy of the unmilled sample was 236.33 kJ/mol, which significantly decreased to 138.97 kJ/mol only by 2 h milling, but a clear marginal diminishing return effect was observed afterward.

**Figure 6:**
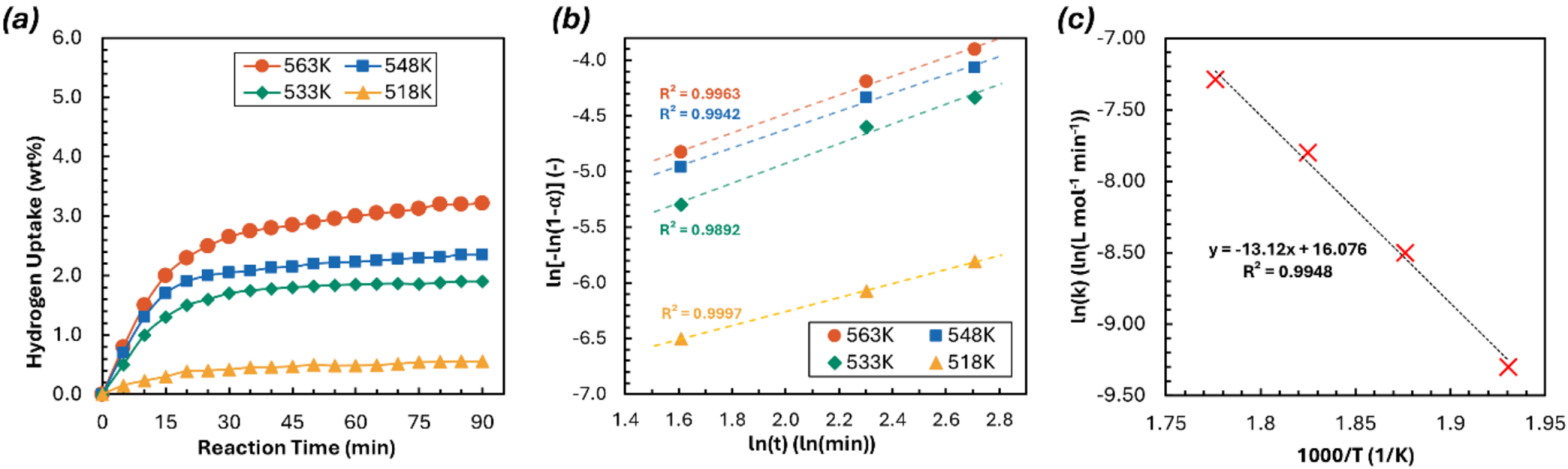
(a) Hydrogen absorption kinetics of Mg_90_-300–2h ball-milled sample at 518, 533, 548, and 563 K, and (b) the corresponding JMAK diagram and (c) Arrhenius plots.

### 3.5 Techno-Economic Analysis

Table 1 presents the approximate energy costs associated with the laboratory-scale production of 10 g of Mg–Ni material, using the eight sample fabrication conditions developed in this study and using pure Mg and Ni powders as starting materials. The amount of hydrogen absorbed by the material in 1 h was used to evaluate the performance of 10 g of material, combining both total capacity and rate of absorption into a single metric. Hydrogen desorption, which reached completion within 10 min for all samples, was not considered. In previous studies, Mulenga and Moys calculated the power consumption of ball mills under various rotational speeds and loading ratios, providing a basis for the milling power estimation in this investigation.^81^

**Table 1:** Energy Consumption and Associated Cost For Production of Mg–Ni Composites, Total Amount of Hydrogen Stored by the Materials after 1 h of Reactions (Estimated from Experiments), and the Corresponding Cost Per Unit H_2_ Capacity^a^

	electricity cost of material production					
	mill power (W)	milling time (h)	electricity cost ($/kWh)	total cost ($)	total H_2_ stored in 1 h (g)	cost per unit H_2_ capacity ($/h·g H_2_)
Mg_90_-300–2 h	675	2	0.32	0.44	0.72	0.61
Mg_90_-300–4 h		4		0.87	0.70	1.25
Mg_90_-300–12 h		12		2.61	0.36	7.26
Mg_90_-300–20 h		20		4.35	0.43	10.08
Mg_90_-400–2 h	900	2		0.58	0.30	1.94
Mg_90_-400–4 h		4		1.16	0.23	5.16
Mg_90_-400–6 h		6		1.74	0.26	6.79
Mg_90_-400–8 h		8		2.32	0.83	2.79

a Energy consumption associated with the production and transport of hydrogen, the production of refined Mg and Ni metal from their ores, and the cooling and heating duties during hydrogen absorption and desorption, was not considered. Wholesale electricity costs for the UK were estimated based on data from Ofgem.^82^

Shorter milling durations and lower rotational speeds resulted in a less costly product as a result of using less electricity during milling while still providing adequate hydrogen storage capacity. For example, the sample showing the highest effective capacity (Mg_90_-400–8h) was able to absorb ∼15% more H_2_ in 1 h than Mg_90_-300–2 h, but at a 460% higher cost per unit H_2_ absorbed, indicating diminishing returns with additional energy expenditure on ball milling. Therefore, in practical operations, a balance must be achieved between low energy consumption during milling, high storage capacity, and fast hydrogen absorption.

## 4 Conclusions

This study evaluated the effects of ball milling on the microstructural and hydrogen storage properties of Mg–Ni composites, focusing on the interplay between milling parameters, structural modifications, and hydrogenation performance, as well as the cost-effectiveness of the different samples produced for practical hydrogen absorption. The impact of ball milling speed and duration showed different effects over a range of physical properties, with competition between particle refinement and agglomeration. For the samples prepared by milling at 400 rpm, longer milling times resulted in considerably faster rates of hydrogen absorption, suggesting that improved Mg–Ni integration, and smaller Mg crystallite sizes, enhanced the catalytic absorption of H_2_ to form bulk MgH_2_. However, estimated operational energy expenditure for different milling conditions indicated that shorter milling durations (2 h) and lower speeds (300 rpm) yielded lower unit overall costs for a given H_2_ capacity, as the improved performance in hydrogen storage from longer milling yielded diminishing returns with respect to energy consumption.

Overall, achieving optimal performance for different application scenarios, whether prioritizing rapid hydrogen absorption/desorption kinetics or maximizing hydrogen storage capacity, requires an appropriate combination of ball milling time and rotational speed, without excessive energy expenditure. Future work should consider a wider range of milling parameters and the effects of altering material composition by adjusting milling procedures to promote formation of mixed Mg–Ni phases. The findings reported here provide insights for the further development of efficient and scalable Mg-based materials for hydrogen storage while highlighting the trade-off between optimal material performance and production cost.

## Supplementary Material



## Notes

Further description of experimental methods and details of hydrogen absorption test rig; measurements of particle sizes; SEM images and EDS maps of samples; collected XRD patterns with fitted Le Bail refinements; N_2_ adsorption isotherms used in BET surface area calculations; total energy contours of milling speed against total milling time; scatter plots showing correlations between microstructural parameters and hydrogen capacity and rate of absorption (PDF)
