# Supplementary material for: Linking the Microstructure of Ball-Milled Mg–Ni Hydrogen Storage Materials to Reactive Properties and Techno-Economic Feasibility
Source: Energy Fuels. 2025 Jul 7;39(28):13789–800. doi: 10.1021/acs.energyfuels.5c01986 (PMC12281568; doi:10.1021/acs.energyfuels.5c01986)
Supplement: Supplementary file 1 [file ef5c01986_si_001.pdf]

# Supporting Information for:
## *Linking the microstructure of ball milled Mg-Ni hydrogen storage materials to reactive properties and techno-economic feasibility*

Haoliang Hong[1], Alexander Harrison[1], Binjian Nie[1*]
[1]Department of Engineering Science, University of Oxford, Oxford, OX1 3PJ, United Kingdom
* Corresponding author: binjian.nie@eng.ox.ac.uk

Section A Further description of experimental methods
Section B Images and measurements of samples
Section C Additional characterization of materials
Section D Contours of milling energy
Section E Plots of all variables with hydrogen storage properties

Section A Further description of experimental methods

Table. S1 Material and ball milling parameters. Sample designations correspond to $Mg_{90}$-[Speed]-[Time]

| Sample Name | Rotational speed during milling (rpm) | Milling time (h) |
|---|---|---|
| $Mg_{90}$-300-2h | 300 | 2 |
| $Mg_{90}$-300-4h | 300 | 4 |
| $Mg_{90}$-300-12h | 300 | 12 |
| $Mg_{90}$-300-20h | 300 | 20 |
| $Mg_{90}$-400-2h | 400 | 2 |
| $Mg_{90}$-400-4h | 400 | 4 |
| $Mg_{90}$-400-6h | 400 | 6 |
| $Mg_{90}$-400-8h | 400 | 8 |

A simple schematic diagram of the Sievert apparatus used to measure hydrogen absorption is shown in Figure. S1.

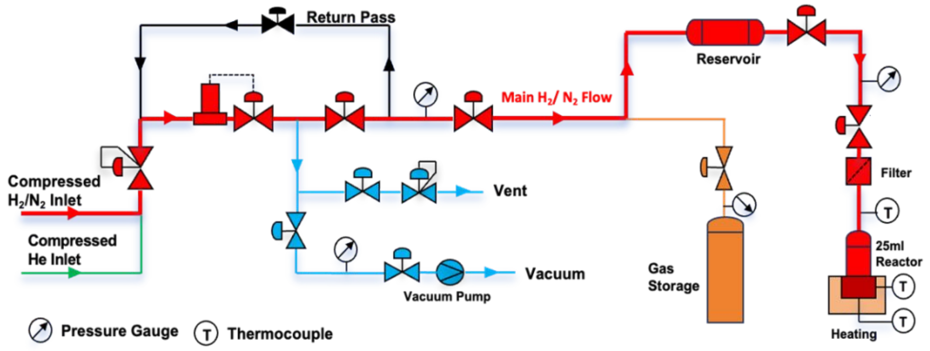


Figure. S1 Schematic diagram showing the main components in the Sievert-type volumetric hydrogen absorption apparatus, used to determine pressure-composition-temperature (PCT) characteristics for materials under investigation.

The system consists of

- Inlet section: Compressed gases ($H_2$, He) are fed into the instrument system through a pressure-reducing valve and a flow controller.
- Gas storage section: Consists of gas storage tanks and reservoirs for filling hydrogen to an initial pressure, and regulating the hydrogen pressure delivered to the reaction section.
- Reaction section: a thermostatic heat source heats a fixed volume reactor to a nominal setpoint temperature. The actual temperature at the surface of the sample is monitored using a K-type thermocouple, and a pressure transducer monitors the overall pressure in the reaction section. A data acquisition system is used to continuously log temperature and pressure measurements at one-second intervals.
- Exhaust section: High pressure gas is discharged into the ventilation pipe after a pressure-reducing valve; low pressure gas (less than or equal to 1 bar gauge pressure) is extracted using a vacuum pump.

All experiments conducted on this platform, including isothermal constant-pressure cycling, temperature-programmed desorption (TPD), and isothermal stepwise PCT measurements, are composed of simple single-pressure-step sorption experiments. The general procedure of this

experiment included sample loading, leak testing, calibration, and hydrogen introduction for the main reaction. Before experiments, all materials underwent a hydrogen absorption and discharge activation process (*i.e.* one additional cycle under experimental conditions) to remove surface oxides and impurities introduced during synthesis, ensuring representative hydrogen storage performance [1].

The Mg-based material was loaded into a 25 mL stainless steel reactor inside a glovebox, ensuring a sealed oxygen-free environment before connecting it to the system. Prior to each experiment, a pressure test was conducted by introducing 10 bar of helium and maintaining for 2 h to confirm the absence of gas leaks. The system was then purged of any residual oxygen or moisture by reducing the system pressure to below $10^{-3}$ bar using a vacuum pump, and repressurising to 5 bar of He, repeated twice.

To calibrate the pressure sensors, the tube furnace was preheated under vacuum for 2 hours to reach a stable temperature. A momentary He introduction (10 bar) followed by rapid valve closure was used to confirm pressure response stability. This technique, vacuum preheating and rapid gas introduction after isotherm, was consistently applied throughout all experiments to minimize thermal fluctuations affecting pressure readings by leveraging the instantaneous adiabatic expansion process [2]. Temperature and pressure variations were then recorded.

In simple single-pressure-step isothermal sorption experiments, once the reaction section had reached the target temperature under vacuum, the valve to the gas storage section was opened, allowing the storage tank to be filled with hydrogen before being sealed. The intermediate valve between the gas storage and inlet sections was then closed. Subsequently, the valve to the reaction section was opened and rapidly closed. The internal temperature and pressure of the reaction zone were continuously recorded for subsequent calculations and analysis.

The hydrogen absorption and desorption properties in PCT curves were conducted after achieving the desired temperature (548 K, 573 K, 598 K and 623 K in this study) under vacuum. The experiment began by introducing $H_2$ at an initial pressure of 0.5 bar, which was then increased stepwise (typically in increments of 1 bar or smaller) by adjusting the hydrogen generator's output pressure, and loading $H_2$ into the gas storage section. When the plateau pressure (*i.e.* the minimum pressure at which reaction began) was reached, hydrogen was then repeatedly injected at the plateau pressure or gradually increased in smaller increments (typically less than 0.1 bar) until the reaction was complete. Afterward, the pressure was further increased following the original stepwise pattern until reaching 20 bar.

The volume of hydrogen absorbed or released by the materials was estimated from pressure measurements using Eq. S-1

$$PV = nZRT \tag{S-1}$$

Where $P$ is the measured pressure (Pa), $V$ is the volume of the reaction section ($cm^3$), $n$ is the number of moles of gas, $Z$ is the gas compressibility (estimated using reported values from the

National Institution of Standards and Technology [3]), $R$ is the universal gas constant (J (mol K)$^{-1}$) and $T$ is the temperature (K).

Assuming constant temperature $T$ throughout the reaction during experiments, the number of moles absorbed during each pressure step was given by Eq. S-2

$$\Delta n = \frac{P_i V}{Z_{i,T} RT} - \frac{P_f V}{Z_{f,T} RT} \tag{S-2}$$

where $Z_{i,T}$ and $Z_{f,T}$ are the hydrogen compressibilities at the temperature $T$ and the initial pressures $P_i$ and final pressure $P_f$, respectively. To complete the multistep PCT curves, the above procedure was repeated for subsequent steps with the values of $\Delta n$ and $P_f$ as a starting point. After $m$ sorption steps, the total hydrogen uptake is given by,

$$n_m = \sum_{j=1}^{m} \left[ \frac{P_{j-1} V}{Z_{T_{j-1}} RT_{j-1}} - \frac{P_j V}{Z_{T_j} RT_j} \right] \tag{S-3}$$

Where $n_m$ is the total change in number of moles of gas (He for calibration and $H_2$ for all experiments here), and $j$ corresponds to each pressure and temperature measurement point (recorded at a rate of 1 $s^{-1}$). To account for the volume occupied in the reactor by the solid material, and the variation in temperature within the reaction section, temperature and volume calibrations were applied, incorporating the sample temperature, system temperature, and the proportion of the heated region. The calibration, and the hydrogen gain and loss were then calculated using Eq. S-4

$$n_m = \sum_{j=1}^{m} \left[ \left( \frac{f P_{j-1}\left(V - \frac{m_s}{\rho_s}\right)}{Z_{P_{j-1},T_{sys,j-1}} RT_{sys,j-1}} + \frac{(1-f) P_{j-1}\left(V - \frac{m_s}{\rho_s}\right)}{Z_{P_{j-1},T_{sample,j-1}} RT_{sample,j-1}} \right) - \left( \frac{f P_j (V - \frac{m_s}{\rho_s})}{Z_{P_j,T_{sys,j}} RT_{sys,j}} + \frac{(1-f) P_j (V - \frac{m_s}{\rho_s})}{Z_{P_j,T_{sample,j}} RT_{sample,j}} \right) \right] \tag{S-4}$$

where $f$ is the percentage volume of the high-temperature isothermal region in reaction section, relative to the total gas volume. The extent of the isothermal region was identified using thermocouples, and the volume then then measured by removing the reactor vessel from the rig, cooling to room temperature, then filling the vessel with water up to the identified point. The reaction section was located downstream of the reservoir in Figure. S1); $m_s$ and $\rho_s$ are the mass and density of the sample; $T_{sys}$ and $T_{sample}$ are the temperature of the system and the sample.

Section B Images and measurements of samples

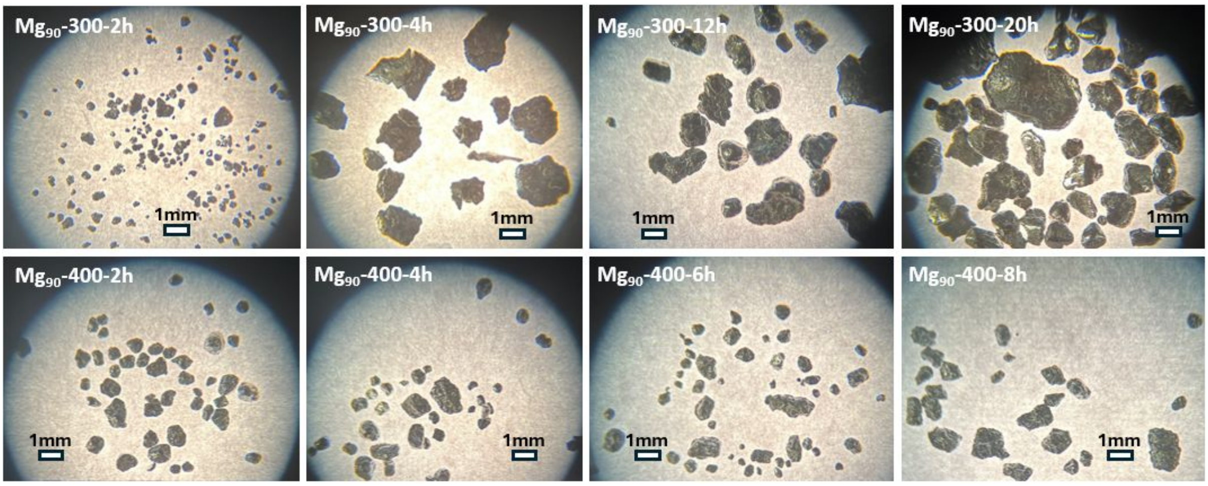


Figure. S2 Brightfield microscope images for eight Mg90wt%-Ni10wt% ball milled samples

Table. S2 Measurements for the average particle size and Sauter mean diameter

| **Sample Name** | **Average Particle Size (um)** | **Sauter mean diameter (um)** |
|---|---|---|
| $Mg_{90}$-300-2h | 316.23 | 455.89 |
| $Mg_{90}$-300-4h | 1439.02 | 2002.99 |
| $Mg_{90}$-300-12h | 1625.79 | 2216.23 |
| $Mg_{90}$-300-20h | 1658.89 | 2266.14 |
| $Mg_{90}$-400-2h | 819.99 | 957.81 |
| $Mg_{90}$-400-4h | 791.75 | 905.63 |
| $Mg_{90}$-400-6h | 602.75 | 863.76 |
| $Mg_{90}$-400-8h | 1105.54 | 1456.59 |

Table. S3 Measurements for the Ni particle size and Sauter mean diameter

| **Sample Name** | **Average Ni Particle Size (um)** | **Sauter mean diameter (um)** |
|---|---|---|
| $Mg_{90}$-300-2h | 7.63 | 14.42 |
| $Mg_{90}$-300-4h | 9.28 | 13.74 |
| $Mg_{90}$-300-12h | 10.82 | 19.88 |
| $Mg_{90}$-300-20h | 9.17 | 15.14 |
| $Mg_{90}$-400-2h | 6.52 | 12.91 |
| $Mg_{90}$-400-4h | 2.68 | 8.07 |
| $Mg_{90}$-400-6h | 3.28 | 10.00 |
| $Mg_{90}$-400-8h | 4.00 | 6.46 |

Section C Additional characterization of materials

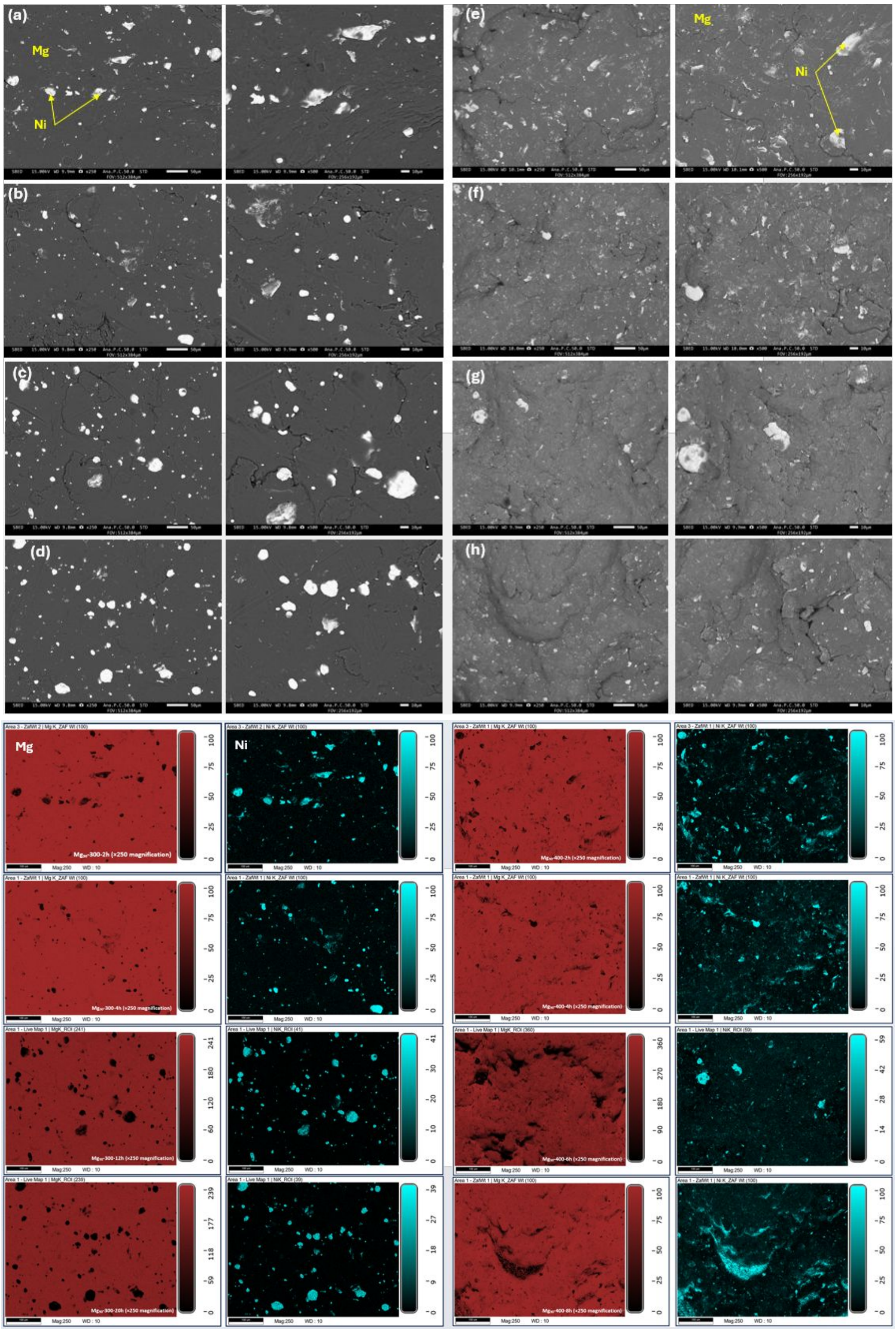


Figure. S3 SEM images of (a) $Mg_{90}$-300-2h (b) $Mg_{90}$-300-4h (c) $Mg_{90}$-300-12h (d) $Mg_{90}$-300-20h (e) $Mg_{90}$-400-2h (f) $Mg_{90}$-400-4h (g) $Mg_{90}$-400-6h (h) $Mg_{90}$-400-8h (each horizontal pair of images correspond to the same sample, ×250 magnification on the left and ×500 on the right) and corresponding EDS maps showing elemental distribution of Mg (red) and Ni (blue) (each horizontal pair of images correspond to the same sample, both ×250 magnification).

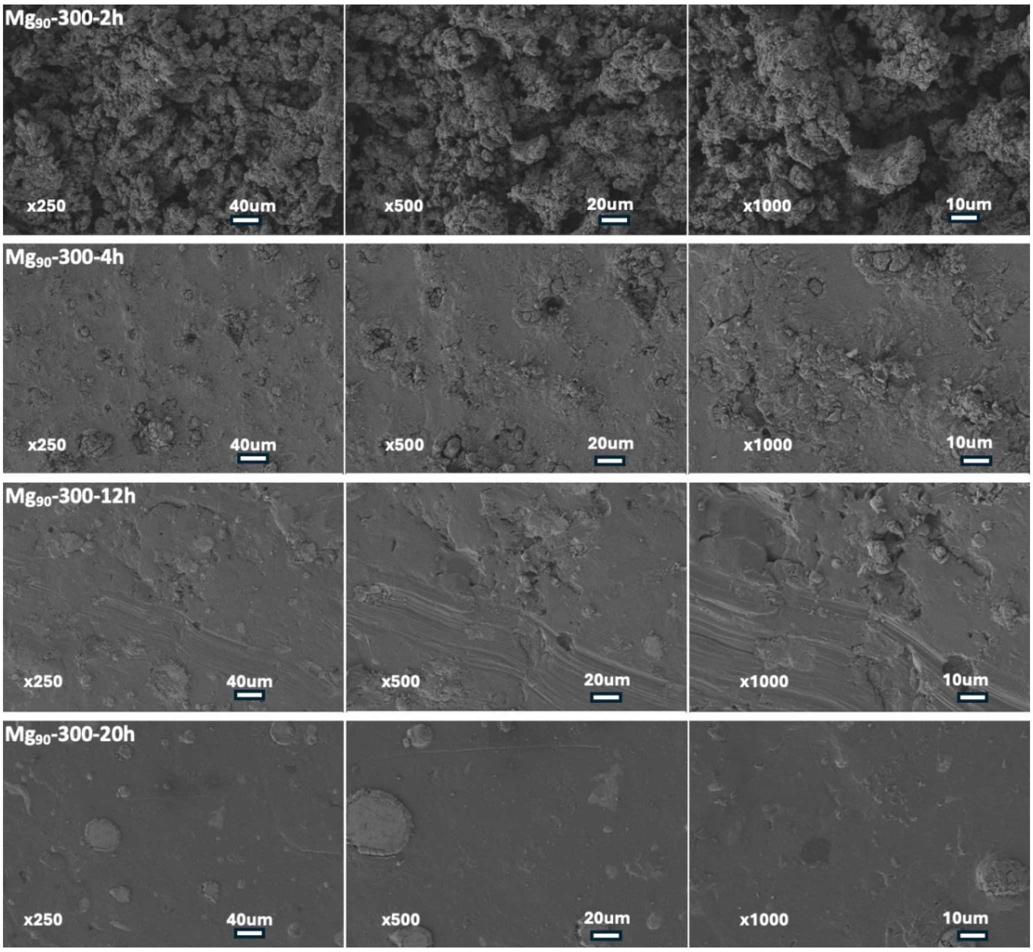


Figure. S4 SEM images of 300 rpm groups after hydrogenation cycles. Each horizontal set of three images correspond to the same sample at different magnification levels.

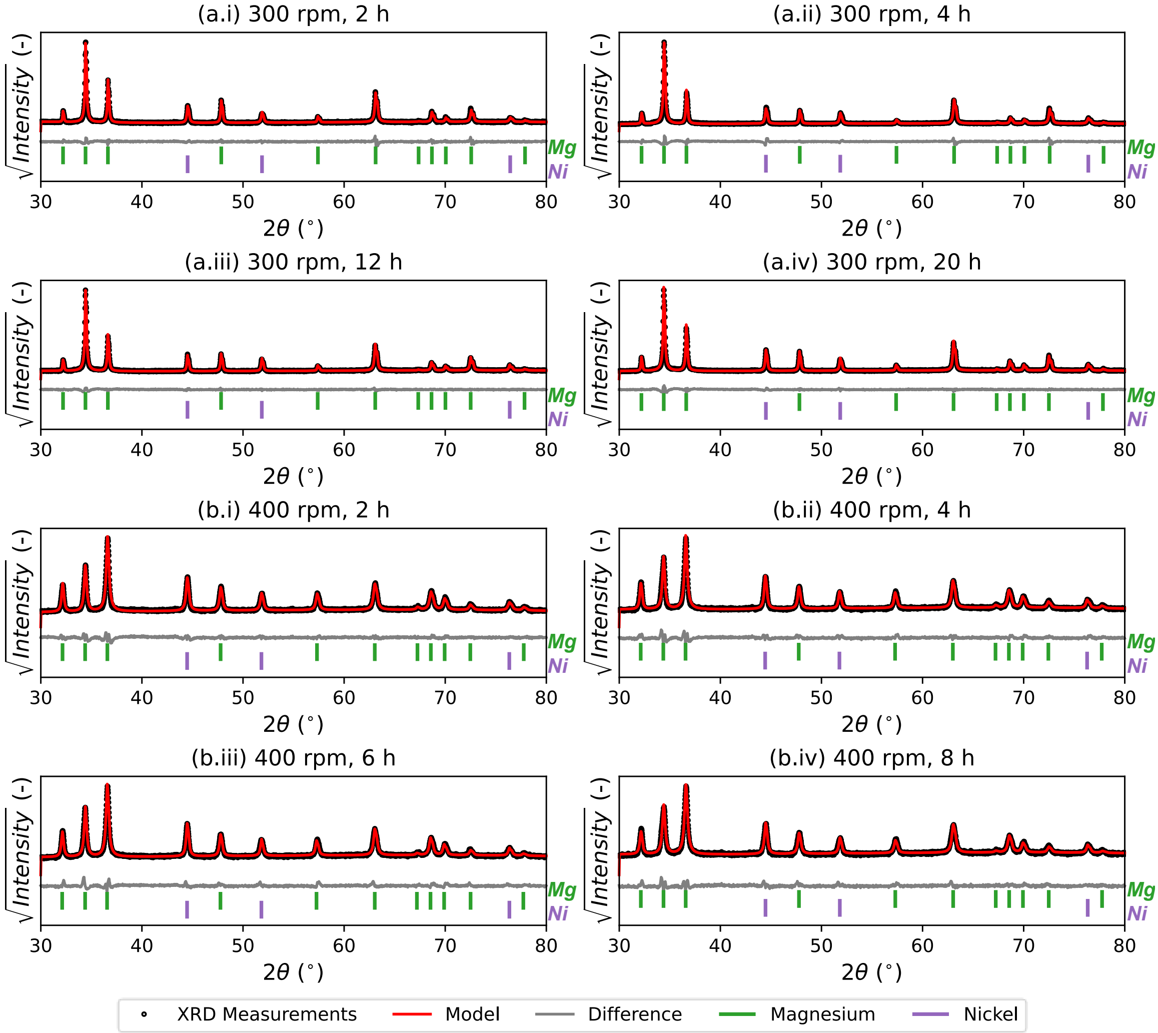


Figure. S5 XRD patterns for eight Mg90wt%-Ni10wt% samples of (a) 300 rpm and (b) 400 rpm. Crosses indicate experimental measurements, solid lines indicate calculated fit from Le Bail refinement, fitted background and difference between fit and experimental measurements. Tick marks indicate fitted peak positions for each phase.

The BET surface area of samples was estimated by measuring the volume of nitrogen adsorbed ($Q$, cm$^3$ g$^{-1}$) over the relative pressure range $\frac{p}{p_0}$ = 0.05-0.25. From BET theory [4,5], the adsorption of a monolayer of nitrogen molecules is given by Eq. S-5

$$\frac{1}{Q\left(\frac{p^0}{p}-1\right)} = \frac{1}{C \cdot Q_{ml}} + \frac{C-1}{C \cdot Q_{ml}}\frac{p}{p_0} \tag{S-5}$$

where $Q_{ml}$ corresponds to the volume of gas adsorbed at a monolayer on the sample (cm$^3$ g$^{-1}$), and $C$ is a fitting constant.

Therefore, plotting $\frac{1}{Q\left(\frac{p^0}{p}-1\right)}$ against $\frac{p}{p_0}$ (shown in Figure. S6) gives a straight line with slope $\beta_1 = \frac{C-1}{C \cdot Q_{ml}}$, and y-intercept $\beta_0 = \frac{1}{C \cdot Q_{ml}}$. Hence, the mass of $N_2$ adsorbed at a monolayer is given by Eq. S-6

$$Q_{ml} = \frac{1}{\beta_0 + \beta_1} \tag{S-6}$$

and the surface area of the sample is given by Eq. C-3, where $S_a$ is the specific surface area ($m^2g^{-1}$), $M_{r,N2}$ is the molar mass of nitrogen (g $mol^{-1}$), $N_a$ is the Avogadro constant ($mol^{-1}$), and $A_{N2}$ is the cross-sectional area of a nitrogen molecule (0.162 $nm^2$)

$$S_a = \frac{Q_{ml} N_a A_{N_2}}{M_{r,N_2}} \tag{S-7}$$

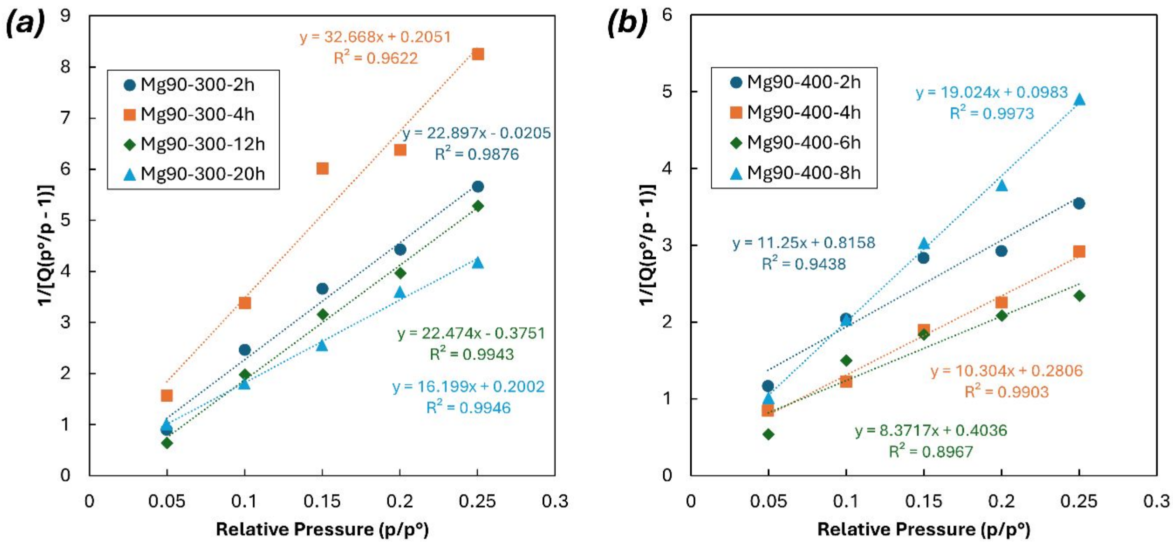


Figure. S6 Linearized nitrogen adsorption isotherms for samples prepared by ball-milling at (a) 300 rpm and (b) 400 rpm milling speed.

Section D Contours of milling energy

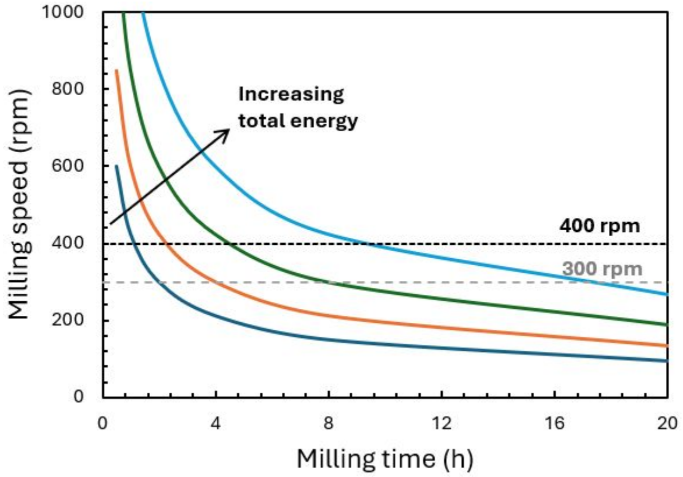


Figure. S7 Contours of constant total energy transfer for variable ball milling time and speed, assuming no energy losses and perfectly inelastic collisions. Dashed guidelines indicate 300 rpm and 400 rpm milling speed as applied in this study.

Section E Plots of all variables with hydrogen storage properties

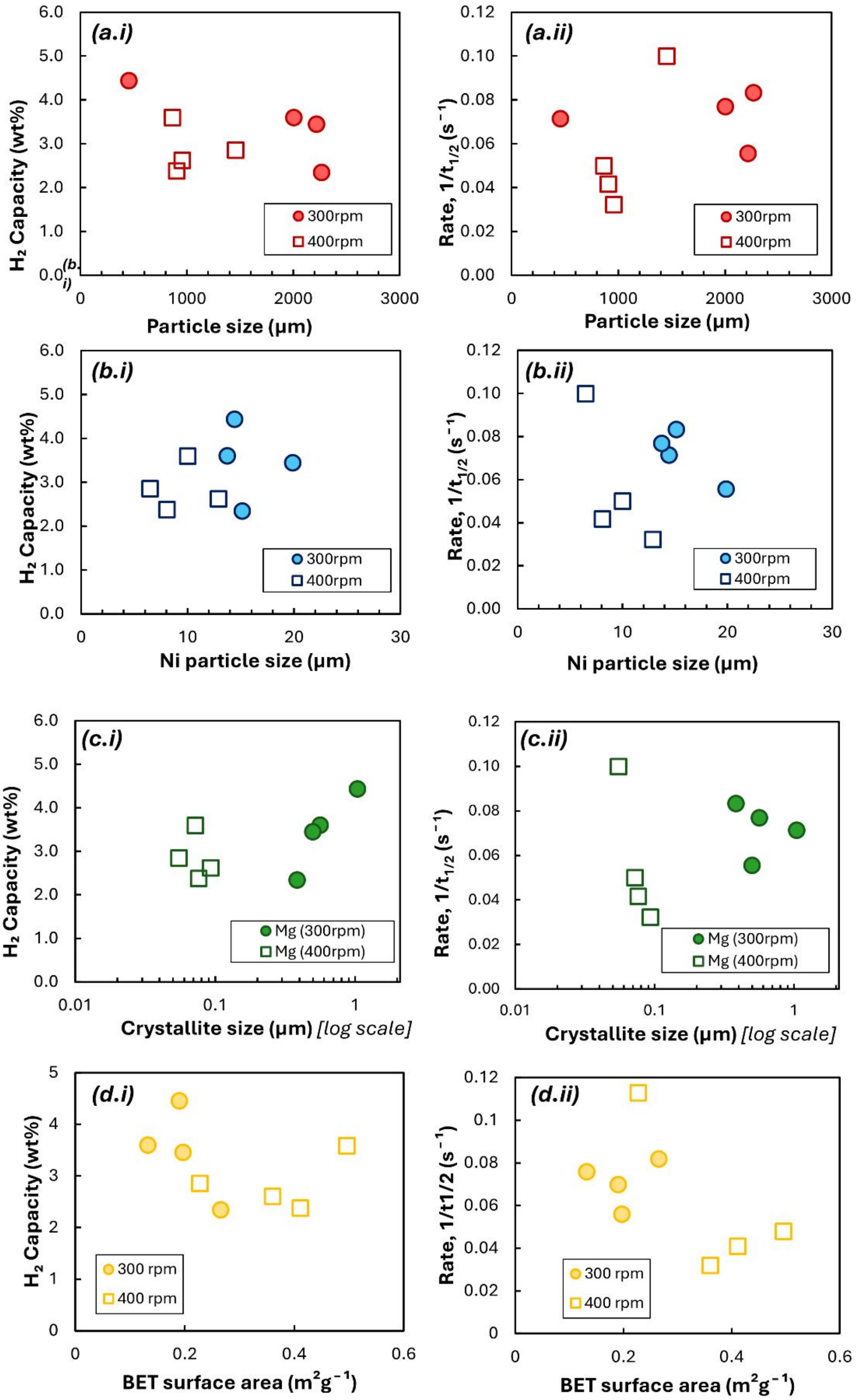


Figure. S8 Relationships between microstructural properties (a) particle size, (b) Ni particle size, (c) Mg crystallite size, and (d) BET surface area, and hydrogen absorption properties (i) total gravimetric capacity and (ii) rate of absorption.

Supplementary References

(1) Yang, H.; Ding, Z.; Li, Y.-T.; Li, S.-Y.; Wu, P.-K.; Hou, Q.-H.; Zheng, Y.; Gao, B.; Huo, K.-F.; Du, W.-J.; Shaw, L. L. Recent advances in kinetic and thermodynamic regulation of magnesium hydride for hydrogen storage. *Rare Metals* **2023**, *42* (9), 2906-2927. DOI: 10.1007/s12598-023-02306-z.
(2) Kanno, H.; Shikazono, N. Experimental study on two-phase adiabatic expansion in a reciprocating expander with intake and exhaust processes. *International Journal of Heat and Mass Transfer* **2016**, *102*, 1004-1011. DOI: https://doi.org/10.1016 j.ijheatmasstransfer.2016.06.081.
(3) Technology, N. I. o. S. a. Charts of Compressibility Factors and Charts Showing Quantities Delivered by Commercial Cylinders for Hydrogen, Nitrogetn, and Oxygen. 1948.
(4) Brunauer, S.; Emmett, P. H.; Teller, E. Adsorption of Gases in Multimolecular Layers. *Journal of the American Chemical Society* **1938**, *60* (2), 309-319. DOI: 10.1021/ja01269a023.
(5) Bardestani, R.; Patience, G. S.; Kaliaguine, S. Experimental methods in chemical engineering: specific surface area and pore size distribution measurements—BET, BJH, and DFT. *The Canadian Journal of Chemical Engineering* **2019**, *97* (11), 2781-2791. DOI: https://doi.org/10.1002/cjce.23632 (acccessed 2025/06/19).
